# An *in-silico* approach to design potential siRNAs against the *ORF57* of Kaposi’s sarcoma-associated herpesvirus

**DOI:** 10.5808/gi.21057

**Published:** 2021-12-31

**Authors:** Anisur Rahman, Shipan Das Gupta, Md. Anisur Rahman, Saheda Tamanna

**Affiliations:** Department of Biotechnology and Genetic Engineering, Faculty of Science, Noakhali Science and Technology University, Noakhali 3814, Bangladesh

**Keywords:** guide strand, human herpesvirus-8, Kaposi’s sarcoma, RNA interference, siRNA

## Abstract

Kaposi's sarcoma-associated herpesvirus (KSHV) is one of the few human oncogenic viruses, which causes a variety of malignancies, including Kaposi's sarcoma, multicentric Castleman disease, and primary effusion lymphoma, particularly in human immunodeficiency virus patients. The currently available treatment options cannot always prevent the invasion and dissemination of this virus. In recent times, siRNA-based therapeutics are gaining prominence over conventional medications as siRNA can be designed to target almost any gene of interest. The ORF57 is a crucial regulatory protein for lytic gene expression of KSHV. Disruption of this gene translation will inevitably inhibit the replication of the virus in the host cell. Therefore, the ORF57 of KSHV could be a potential target for designing siRNA-based therapeutics. Considering both sequence preferences and target site accessibility, several online tools (i-SCORE Designer, Sfold web server) had been utilized to predict the siRNA guide strand against the ORF57. Subsequently, off-target filtration (BLAST), conservancy test (fuzznuc), and thermodynamics analysis (RNAcofold, RNAalifold, and RNA Structure web server) were also performed to select the most suitable siRNA sequences. Finally, two siRNAs were identified that passed all of the filtration phases and fulfilled the thermodynamic criteria. We hope that the siRNAs predicted in this study would be helpful for the development of new effective therapeutics against KSHV.

## Introduction

Kaposi’s sarcoma (KS) is a multifocal hyperproliferative neoplasm of the vascular or lymphatic endothelium. KS lesions may also occur on the skin surface of the upper and lower extremities or on the visceral organs like the lung and spleen [1-4]. Four epidemiological subtypes (Classic, Endemic, Epidemic, and Iatrogenic) of KS have been described so far and Kaposi’s sarcoma-associated herpesvirus (KSHV), also known as human herpesvirus-8 was identified as the primary etiologic agent of these subtypes [5-7]. Besides, KSHV causes two lymphoproliferative diseases and an inflammatory syndrome: primary effusion lymphoma, multicentric Castleman disease, and KSHV inflammatory cytokine syndrome [8-10].

KSHV has the highest prevalence in sub-Saharan Africa, the intermediary prevalence in the Mediterranean, and low prevalence in northern Europe, North America, and Asia [4]. Previously, KS was 20,000 times more persistent in acquired immune deficiency syndrome (AIDS) patients than in the general population [11]. With the advent of combination antiretroviral therapy, the incidence of AIDS-related (epidemic) KS substantially decreased in developed countries [12]. But the rapid progression of AIDS and inaccessibility to antiretroviral drugs worsened the condition in several African countries [13]. Countries like Malawi, Uganda, Zimbabwe, and Swaziland have experienced a multitude of KS incidences since the onset of the AIDS epidemic in the early 1980s, making KS the most common cancer in males and the second most common cancer in females [14,15].

The KSHV genome has approximately 140.5 kb long unique region (LUR) flanked by 25‒30 kb of direct terminal repeats [16,17]. The LUR comprises 90 open reading frames (ORFs) that separately express during the latent or lytic phase of viral infection [18]. Though ORF50/Rta (replication and transcription activator) initiates the entire KSHV lytic cycle, its completion depends on ORF57 that ensures the orderly expression of other early and late lytic genes [19]. The ORF57/Mta (mRNA transcript accumulation) is a 455 amino acid long nuclear protein with no substantial sequence homology to any known cellular proteins [20]. This post-transcriptional regulator plays several vital roles in KSHV pathogenesis, for example, binding and stabilization of intron-less transcripts, facilitates intron splicing, nuclear accumulation of coding/non-coding RNAs, cytoplasmic accumulation of mRNAs, promoting protein translation, etc. [21]. Disruption of *ORF57* showed to interrupt the KSHV lytic gene expression and eventually halt virion production [22]. Therefore, it could be a potent target for therapeutic intervention to suppress the replication of KSHV in the host cells.

RNA interference is a unique molecular therapeutic technique in which specific non-coding RNAs, such as siRNA or miRNA, silence or downregulate the target mRNA in a sequence-specific manner. Although siRNA and miRNA share a common pathway, they have distinct functions, and in many cases, siRNA outcompeted miRNA in silencing activity. Since siRNA designing allows more sophistication in reducing off-target effects, it can inhibit one particular target mRNA. Besides, modern transfection techniques such as lipofection and nanoparticles have greatly simplified its delivery into cells [23]. siRNA-based treatment has been successfully demonstrated in influenza, hepatitis C, severe acute respiratory syndrome coronavirus 2, cancer, different autoimmune, and neurodegenerative diseases [24-28]. Hence, considering the clinical significance of KSHV, the role of ORF57 in viral pathogenesis, and the therapeutic potentiality of RNA interference, we aimed in the present study to design effective siRNA(s) against *ORF57* of KSHV.

## Methods

**Fig. 1.**
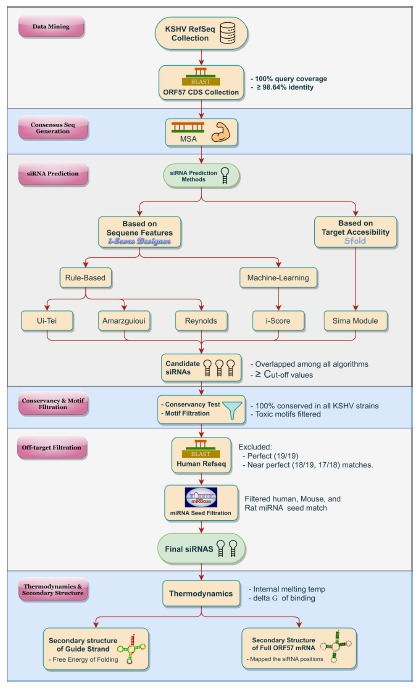
Schematic diagram of the overall methodology. Designing of effective siRNA/(s) against the ORF57 of Kaposi's sarcoma-associated herpesvirus (KSHV) was divided into several phases. After initial sequence data collection and representative consensus sequence generation for ORF57, five distinct algorithms were adopted for siRNA prediction. Final siRNAs were selected through a rigorous filtration process (conservancy, toxic motif, off-target, miRNA seed). The secondary structure and thermodynamic properties of each siRNA were also evaluated. CDS, coding sequence; MSA, multiple sequence alignment.

The overall pictorial representation of this study is depicted in Fig. 1 in a stepwise manner.

### Data mining

The coding sequence (CDS) of *ORF57* was obtained from the KSHV reference sequence (NC_009333.1) in the NCBI GenBank (https://www.ncbi.nlm.nih.gov/genbank/) database [29]. This reference CDS was used as a query in the NCBI nucleotide BLAST tool (http://www.ncbi.nlm.nih.gov/blast) to identify all the available CDS of *ORF57* of KSHV [30]. During BLAST search, non-redundant nucleotide collection (nr/nt) was selected as a database and the search was restricted to only KSHV sequences by selecting txid: 37296 in the organism section. The maximum number of target sequences was set to 1,000 and other parameters were kept as default. BLAST analysis resulted in a total of 76 sequences with 100% query coverage and a percent identity ≥ 98.64%. The accession numbers of these 76 sequences were retrieved, and the CDS of *ORF57* from each accession were downloaded from NCBI using command-line code.

### Generation of consensus sequence

In the UGENE v34.0 software [31], the CDS of all collected *ORF57* were subjected to multiple sequence alignment (MSA) by the MUSCLE [32] tool with default parameters. UGENE employs JalView [33] as a default algorithm to generate a consensus sequence from MSA. The consensus sequence constructed from this MSA analysis was considered to be the representative sequence of the *ORF57* of all KSHV strains.

### Prediction of siRNA

The consensus sequence obtained from MSA was used to predict potential siRNAs against the *ORF57* gene. In this regard, two parameters (e.g., sequence features and target site accessibility) were taken into consideration [34]. The i-SCORE Designer web tool (https://www.med.nagoya-u.ac.jp/neurogenetics/i_Score/i_score.html) has been deployed for sequence-based siRNA design [35]. This tool computes nine distinct algorithm scores (Ui-Tei, Amarzguioui, Hsieh, Takasaki, s-Biopredsi, i-Score, Reynolds, Katoh, and DSIR) for siRNA prediction by analyzing different nucleotide preferences from target mRNA.

Sequence-focused algorithms can further be subdivided into two groups (rule-based and machine-learning aided) depending on their calculation nature [34]. Ui-Tei, Amarzguioui, and Reynolds scoring schemes had been taken into account for this study as rule-based approaches. The conditions of those rules are listed in Supplementary Table 1. The i-Score (inhibitory score) algorithm, which implements a linear regression model to predict siRNAs, was employed for machine-learning aided siRNA designing. This method solely examines nucleotide preferences at each position to estimate the score. Finally, the Sirna module of the Sfold web server (http://sfold.wadsworth.org) was used to identify putative siRNAs based on target accessibility [36]. But the module also incorporates the sequence rules as well as accessibility criteria [37].

Only the results that scored equal to or greater than the recommended cutoff values of each of the five algorithms (Table 1) were taken. The siRNAs that overlapped across all five algorithms were then selected for subsequent analysis.

### Conservancy test and motif filtration

Every selected siRNA target was tested for 100% conservancy among all KSHV strains. In the next step, the occurrence of some sequence motifs (“UGUGU,” “GUCCUUCAA,” “AUCGAU(N) nGGGG,” “UGGC”) was screened for every siRNA candidate. Because such motifs are suggested to be immune-stimulatory by some experimental evidence and should be avoided during the design of siRNA [41]. These steps were conducted by employing the *fuzznuc* command-line tool from EMBOSS software package [42].

### Filtration of off-target sites

A two-step filtration process was performed to assess the likelihood that candidate siRNAs would cause off-target effects. In the first step, the NCBI nucleotide BLAST was employed to screen a perfect (19/19) or near-perfect (18/19, 17/19) match of the human RefSeq mRNA database against both sense and antisense strands of candidate siRNAs [43,44]. BLAST's default parameters are inefficient for evaluating very short sequences of siRNAs, thereby, some parameters have been customized according to the guidelines reported in Birmingham et al. [45]. The BLAST options used for this study to identify off-targets are listed in Supplementary Table 2. siRNAs showing complete or nearly complete complementarity with off-target mRNA are assumed to have off-target effects, and therefore have been rejected.

In the second step of the off-target screening, the seed region (2nd to 7th nucleotide from 5' end) of the selected siRNAs was compared to the seed region (2nd to 8th nucleotide from 5' end) of miRNAs found in humans, mice, and rats. All the human, mouse, and rat miRNAs have been downloaded from miRBAse (http://www.mirbase.org/index.shtml), a publicly accessible microRNA database of annotated miRNA sequences [46]. The siRNAs, whose seed region matched with the seed region of human, rat, and mouse miRNAs were eliminated in this phase.

### Thermodynamic analysis

The internal melting temperature (Tm) of the sense strand of each candidate siRNA duplex was calculated by the OligoEvaluator analysis tool (http://www.oligoevaluator.com). To estimate the free energy of heterodimer binding (ΔG) between target mRNA and siRNA guide strand RNAcofold web server (http://rna.tbi.univie.ac.at/cgi-bin/RNAWebSuite/RNAcofold.cgi) was used with defaults parameters [47]. This program calculates the base-pairing pattern and hybridization energy of interacting RNA strands by utilizing thermodynamic and kinetic properties. To estimate base-pairing probabilities it implements an extension of McCaskill’s partition function algorithm [48]. The frequency of the minimum free energy (MFE) structure in the ensemble and ΔG for heterodimer binding was calculated for every candidate siRNA duplex (sense and antisense strand).

### Determination of secondary structure of guide strand and free energy of folding

The MaxExpect algorithm of the RNA Structure web server (https://rna.urmc.rochester.edu/RNAstructureWeb/) has been employed to appraise the secondary structure of the guide strand [49]. In addition to the structure prediction, the MaxExpect program produces CT files for each structure. The CT file obtained for each of the siRNA guide strands was then used as input for another RNA Structure web server tool called efn2. This efn2 algorithm calculates the folding free energy change of the secondary structure of the guide strand from the CT file [50].

### Prediction of the secondary structure of full mRNA and mapping target sites

RNAalifold server from Vienna RNA Web Services has been employed to predict the secondary structure of *ORF57* mRNA [51]. This server generates a consensus secondary structure from the alignment of multiple related DNA or RNA sequences. The MSA file, previously generated by the MUSCLE algorithm, was supplied as input in this phase. The new RNAalifold with the RIBOSUME scoring option was chosen as the RNAalifold version. Besides, other settings were kept in the default option. This tool provides an MFE structure that was downloaded in Vienna format. Vienna RNA Web Service also has a visualization tool called *forna* server that can be used to design and import RNA secondary structures interactively [52]. The secondary structure of full *ORF57* was drawn on this server with the Vienna file retrieved from the previous tool. In addition, the target sites of the chosen siRNAs have been mapped within the structure of this full mRNA.

## Results

### Prediction and selection of siRNA

After performing BLAST using CDS of *ORF57* obtained from KSHV RefSeq, we got *ORF57* sequences of 76 strains and isolates of KSHV for subsequent analysis. Accession numbers of all selected strains and isolates are listed in Supplementary Table 3. A 1.3 kb long consensus sequence was generated from the 76 *ORF57* sequences using MSA of the MUSCLE tool (see Supplementary Table 4). Following a systematic screening, twelve candidate siRNAs were identified that scored at or above the recommended threshold value for each of the five algorithms specified above (Supplementary Table 5).

Out of these twelve, eight siRNAs displayed 100% conserved target sequences across all KSHV strains. The next step was to eliminate siRNAs with immune-stimulatory motifs (GUCCUUCAA, UGUGU, AUCGAU, and UGGC) in their guide strands. Two of our siRNA guide strands were found to have the “UGGC'' motif and thereby excluded from the candidate list. Though there is no clear evidence, some studies suggest avoiding siRNA candidates with low complexity motifs such as “AAAA”, “CCCC”, “GGGG”, or “UUUU” [41]. The Sfold algorithm filters out such guide strands in the case of siRNA duplexes with a total score of 12 or higher. The siRNAs that have passed the filtration steps so far have been named from siRNA_1 to siRNA_6 for ease of exposition (Supplementary Table 6).

For off-target filtration, BLAST analysis was performed with both strands of candidate siRNAs against the human genome to filter out the undesired siRNAs. Two siRNA duplexes (siRNA_3 and siRNA_6) were filtered out and excluded from the final list as those possessed nearly identical (17/19 and 17/18) sequence segments in respect to the human genome (Supplementary Table 7). In the next phase of off-target filtration, siRNA_2 and siRNA_5 were excluded from the candidate list as their seed regions were shown to be similar to those of human miRNA seeds (Supplementary Table 8). There are differing opinions on the position and length of the seed region of miRNA (6‒8 mers), but in higher mammals, the seed region is regarded to be in the 2nd to 8th position (7-mers) from the 5' end [45,53]. Therefore, in order to be on the safe side, the 2nd to 8th nucleotide (7-mers) of miRNA was considered as the seed region. Our recommended siRNA_1 and siRNA_4 satisfied all of the sequence properties and filtration conditions of this study. Eventually, siRNA_1 and siRNA_4 were found to meet all of the sequence properties and filtration conditions and hence selected as final siRNAs (Table 2). It is noteworthy to mention that, seed region of siRNA_4 matched with a mouse miRNA seed.

The percentage of GC content in each siRNA was also noted because low GC content can result in poor and nonspecific binding, whereas high GC content prevents the helicase and RNA-Induced Silencing Complex (RISC) complex from unwinding the siRNA duplex [54]. Many studies have proposed various acceptable GC content limits [38,39]. Considering all nucleotide preferences, Fakhr et al. [55] suggested that the GC content of siRNA should be between 36%–52%. All of the siRNAs selected in this study have a GC content within this range.

### Thermodynamic attributes

The silencing machinery of siRNA is largely modulated by the thermodynamic stability of nucleotide base pairing [56]. The internal melting temperature (Tm) and free energy change (∆G) between siRNA seed and mRNA target are reliable markers of the thermodynamic stability of such heteroduplexes [57]. Different thermodynamics properties of the selected siRNAs are listed in Table 3. Both siRNAs were found to have internal melting temperatures (Tm) below 60°C. The free energy of heterodimer binding between siRNA and target mRNA is the consequence of two energy contributions. The first one is the energy used to open the binding site, and the other is the energy obtained from hybridization. The RNAcofold web server calculates the free energy of heterodimer binding (∆G) as per the following equation:

∆G_Binding_ = ∆G_AB_ ‒ ∆G_A_ ‒ ∆G_B_ [47].

**Fig. 2.**
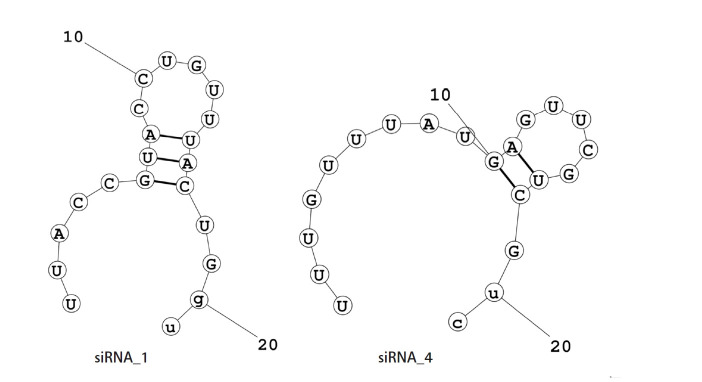
Folding structure of guide RNAs. Potential intra-oligomer binding within the secondary structure of siRNA guide strands. The free energies for the folding structures of siRNA guide strands were also generated from these secondary structures. siRNA guide strand with the positive free energy of folding has a higher probability of binding with the target mRNA. Here, the positive free energy of the siRNA_1 and siRNA_4 for the folding structure of their guide strands are 0.3 kcal/mol and 0.5 kcal/mol, respectively.

**Fig. 3.**
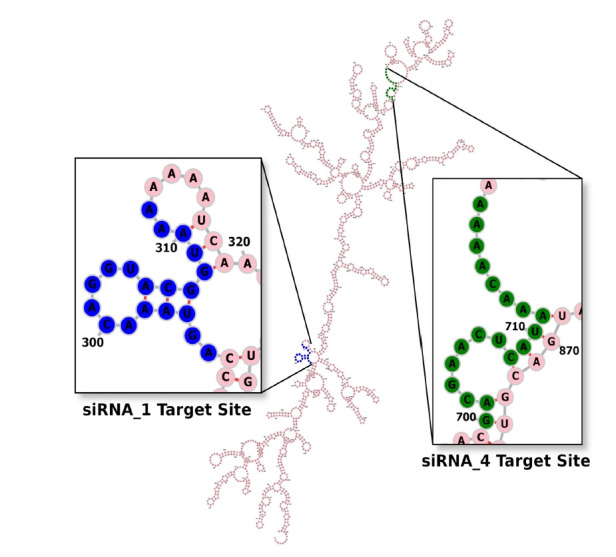
Secondary structure of the target mRNA. From the multiple sequence alignment file generated by the MUSCLE tool, the RNAalifold server produced a consensus secondary structure of the entire ORF57 mRNA of Kaposi’s sarcoma-associated herpesvirus. Target sites of both siRNA_1 and siRNA_4 (highlighted in blue and green color, respectively) seem to have an unpaired 5′ or 3′ end, which will favor their silencing activity.

The net ∆G value of the mRNA-siRNA duplex should be negative for better interaction. The higher the negative ∆G value, the more stable the duplex will form between siRNA and target mRNA [58]. But for proper siRNA silencing effect, this ∆G value should not be too high or too low, and a ∆G value between ‒35 and ‒27 kcal/mol yields better performance [59]. The values of ∆G for siRNA_1 and siRNA_4 were ‒29.16 kcal/mol and ‒27.42 kcal/mol, respectively. In the ensemble of secondary structures, the MFE structure of both siRNAs seemed to have a relatively higher frequency (50.54% for siRNA_1 and 42.73% for siRNA_4). The free energy for the folding of the selected siRNA guide strands was also calculated, along with their probable folding structures (Fig. 2). Both siRNA_1 and siRNA_4 tended to fold with a positive free energy (0.3 kcal/mol and 0.5 kcal/mol, respectively). Then, the selected siRNAs were ranked based on their off-target and thermodynamic properties, with siRNA_1 being the first and siRNA_4 in the second position. Finally, target sites of the two selected siRNAs were mapped onto the secondary structure of this mRNA (Fig. 3).

## Discussion

There are several algorithms, standalone tools, and web servers available for designing siRNA against target sequences, each with its own set of benefits and drawbacks [55]. These tools consider a diverse array of mechanisms and features to screen out the best possible siRNAs, and yet none of them can adopt the all-in-one strategy. That’s why a somewhat manual hybrid approach was employed in this study. We utilized a combination of different algorithms (Reynolds, Amarzguioui, Ui-Tei, i-Score, and Sfold) to acquire siRNAs that met most of the prerequisites to be an effective gene silencer. For example, Reynolds, Amarzguioui, Ui-Tei algorithms are some of the most widely used rule-based algorithms, and many tools implement these algorithms for initial siRNA prediction [34,41,60,61]. Second-generation algorithms like i-Score, s-Biopredsi, and DSIR implement different machine-learning methods to predict the most promising siRNAs with almost equivalent accuracy [35,60]. On the other hand, Sfold uses a statistical algorithm-based probability profile approach to predict siRNA accessible regions in the secondary structure of target mRNA [36]. While other algorithms perform calculations based on 19-mer siRNA sequences, s-Biopredsi and DSIR consider 21-mer for their analysis [35]. That’s why from the second-generation category, only the i-Score algorithm was taken into account to ensure uniformity in siRNA length and computation. Only those siRNAs were picked from all of the predicted siRNAs that met every key criterion of these five methods. Furthermore, the full conservation of these siRNA target sites was also verified to ensure that our proposed siRNAs are effective against all KSHV strains.

Previously it was reported that siRNA sequences with “GUCCUUCAA” and “UGUGU” motifs can be immune-stimulatory. For example, the “UGUGU” motif induces IFN type 1 and causes downregulation of nonspecific genes [62,63]. Another experiment demonstrated that monocytes are stimulated to generate large amounts of IL-12 when the CpG motif "AUCGAU" is present in RNA oligonucleotides along with a poly-G tail [64]. The existence of the "UGGC" motif in the siRNA guide strand has been found to decrease cell viability [65]. The siRNAs which contained these sorts of motifs were omitted in this phase of screening.

After the conservancy and motif filtration analysis, both the sense and antisense of the selected siRNAs were investigated for the presence of off-target human gene and miRNA seed regions. The siRNA-targeted sequence of viral mRNA should not be identical or nearly identical to any human gene, otherwise, it will create an undesired silencing effect [66]. Off-target effects can be induced by either strand of siRNA (sense or antisense) [45]. Furthermore, siRNA guide strands with seed regions identical to the human miRNA were sieved out in the second phase of off-target filtration. Because miRNA-like off targeting may induce mRNA translation inhibition, which can also result in gene-nonspecific downregulation [67].

For effective siRNA activity, target sites should not be located in SNP sites, and/or in between the first 75 bases of mRNA's start codon or in intron sites [55]. Since all of the target sites for the candidate siRNAs of this study are fully conserved across all mRNA sequences, it can be assured that these target sequences are not in SNP sites. None of the selected siRNAs' target sites are within the first 75 bases of mRNA. Finally, the use of only coding sequences in this analysis eradicated any possibilities of the intron sequences being found in target sites (Table 2). GC content of siRNA has a significant impact on its silencing efficacy, and our suggested siRNAs have GC content in the recommended range [55].

The performance of siRNA is influenced not only by sequence features but also by structural and thermodynamic properties [68]. The incorporation of RNA strands in the RISC complex can be influenced by the thermodynamics of the siRNA duplex. More specifically, RNA strands having lower binding stability at the 5' end of the guide strand are preferred to be integrated into the RISC. Furthermore, the thermodynamic features of nucleotide base-pairing between the siRNA guide strand seed region and off-target mRNAs are primarily responsible for its off-target silencing activity [69]. Therefore, after selecting the desired siRNAs through a stringent filtration procedure, their thermodynamic attributes were also evaluated. The existence of hairpin structures in target mRNA sites can be anticipated by calculating the internal melting temperature (Tm) of the sense strands. Especially, if the Tm value of the siRNA sense strand is greater than 60℃, there is a high probability of hairpin formation, which will eventually reduce the knockdown ability [70]. None of our suggested siRNAs exceed this threshold value of Tm (Table 3).

One of the most essential factors for implementing siRNA target prediction algorithms is the computational estimation of RNA-RNA binding interaction. This RNA-RNA binding interaction is indicated by the free energy of binding (∆G) between a specific siRNA and its target mRNA. In case of weak duplex (mRNA-siRNA) stability, the RISC would not have enough opportunity to cleave the target mRNA. Contrarily, the passenger strand or target mRNA will separate slowly after cleavage by the RISC complex if the siRNA forms a very stable duplex with its antisense strand. All of the finalized siRNAs in this study have free energy values of heterodimer binding (∆G) in between the recommended range (Table 3).

Another important indicator of the strength of base-pairing in a seed duplex complex is the frequency of MFE structure in the ensemble of secondary structures. A high frequency of MFE structure in the ensemble is correlated to a stiff seed duplex binding [71]. The accessibility of siRNA for binding, which can be projected by its folding free energy change, is found to be highly correlated with its efficacy for hybridization with target mRNA [72]. According to prior findings, an RNA molecule with the positive free energy of folding has a greater likelihood of binding to a target site because it will be more accessible [73]. Furthermore, intra-oligomer binding within the antisense strand of siRNA can significantly impede its accessibility for target mRNA [72]. That's why potential folding structures of selected siRNA guide strands were also predicted in this analysis (Fig. 2). Both siRNAs reported in this investigation (siRNA_1 and siRNA_4) appeared to have a high frequency of MFE structure and positive free energy of folding (Table 3).

Aside from choosing a siRNA guide strand, predicting the secondary structure of the target mRNA is also very imperative for RNAi activity. The previous evidence indicated that the secondary structure of complete mRNA should be postulated, as nucleotides distal from the target site can also modify its (target site's) structure [74]. But the number of potential secondary structures increases exponentially as the length of the sequence increases, making reliable mRNA secondary structure prediction challenging [72]. For that reason, the RNAalifold server, which predicts the consensus secondary structure of mRNA from MSA among several related sequences, was used in this experiment. Target mRNA with unpaired regions at either 5’-end or 3’-end can be silenced more effectively than a fully paired target [75]. As shown in Fig. 3, the target sites of our selected siRNAs contain an unpaired 5' or 3' end.

*ORF57* of KSHV plays a vital role in viral lytic replication. Dimerization of *ORF57* stabilizes the protein structure and is crucial for its functional activity. Each monomer of *ORF57* homodimer is encoded from a single gene and consists of two distinct domains: intrinsically disordered N-terminal domain (NTD) with no defined structural motifs (amino acid residues 1‒166), and helix-rich C-terminal domain (CTD) (amino acid residues 167‒455) [76]. The NTD harbors three nuclear localization signals (NLSs) and interacts with several cellular factors [20,77]. The CTD has an N-terminal ‘arm’ stretching from residues 167 to 222 and a C-terminal globular domain (aa residues 223‒455) having a conserved zinc-binding motif. These three structural elements (arm, globular interface, and zinc-binding motif) are equally significant for *ORF57* dimerization as the ‘arm’ region from one monomer docks on the globular surface of a neighboring monomer in an antiparallel fashion, whereas the C-terminus end (aa residues 445‒454) is locked into the globular domain of the same monomer [76].

Interestingly, both the siRNA_1 and siRNA_4 target regions are located in the CDS of N and C-terminal domains of *ORF57*, respectively. Three NLSs are found in the NTD and the siRNA_1 target sequence (aa residues 98-104) overlaps with the NLS1 (aa residues 101‒107). A previous study demonstrated that disruption of NLSs inhibits the nuclear translocation process of *ORF57* [77]. Our designed siRNA_1 targets the NLS1 coding region and could potentially disturb the translation process of the NTD CDS of *ORF57*. Besides, *ORF57*-CTD facilitates the dimerization, stability, and function of the protein. Functional studies also revealed the dissociation of *ORF57* dimer upon deletion/point mutation of either one of three structural elements as mentioned earlier. siRNA_4 designed in this study falls within the CDS of the globular interface (aa residues 234‒239) of *ORF57*-CTD. This globular structure maintains the electrostatic interaction with the interface residues of CTD of adjacent monomer to stabilize the dimer [76]. Thus, our proposed siRNA_4 could also inhibit this function by degrading the target mRNA.

It is well established that siRNA-induced post-transcriptional gene silencing starts with the assembly of the RISC [78,79]. The mRNA molecule is cut exactly by cleaving the phosphodiester bond between the target nucleotides which are paired to siRNA residues [80]. The functional ORF57 protein is a dimer of two identical subunits. This indicates the subunits of ORF57 protein are translated from the transcripts of a single copy gene. Hence, the disruption of the *ORF57* transcripts by siRNAs could stop the translation process completely. Therefore, we believe that either siRNA_1 or siRNA_4 could be sufficient enough to suppress the *ORF57* gene expression completely or at least partially.

There is currently no specific treatment for KSHV-related diseases. The treatment of choice for KSHV patients predominantly depends on various parameters, like the tumor location, a variant of KS, rate of progression, distribution of the lesions, the severity of the symptoms, and immune competence [81]. Although medicines such as rituximab, acyclovir, and others are currently used to treat KSHV-related complications, these are not specific therapies [82,83]. Besides that, severe side effects like kidney toxicity, neutropenia, and neurotoxicity have made the treatments more challenging [84]. That’s why additional studies are required to explore new drugs for KSHV associated diseases. In this circumstance siRNA-based therapy might be a viable alternative as the inhibitory effect of siRNA on different herpes virus replication has already been reported in several studies [85-87]. This research is such an effort to accelerate the discovery of new treatments for KSHV-related diseases. Two potential siRNAs have been screened in this study through a series of comprehensive filtration steps that will hopefully inhibit the translation of the *ORF57* gene in KSHV. Since our suggested siRNAs meet all of the requirements for an effective siRNA, it can be expected that they'll be able to inhibit the infection against all KSHV strains. But, as this selection method was entirely based on computational prediction, proper in vitro and in vivo validation is albeit necessary.

## Figures and Tables

**Table 1.** siRNA design algorithms and their cutoff values

Algorithms	Cutoff-value	Reference
Based on sequence features		
Rule-based		
Reynolds	≥6	[38]
Amarzguioui	≥3	[39]
Ui-Tei	Ia & Ib	[40]
Machine learning approach		
i-score	≥66	[35]
Based on target accessibility		
Sfold Sirna module	≥12	[37]

**Table 2.** Final siRNAs

Rank	Name	Start positions	Sense	Antisense	GC (%)
1	siRNA_1	294	CAGUAAACAGGUACGGUAA	UUACCGUACCUGUUUACUGgu	42.1
2	siRNA_4	700	CGACGAACUCAUAAACAAA	UUUGUUUAUGAGUUCGUCGuc	36.8

**Table 3.** Thermodynamics results

Rank	Name	Internal melting Tm (℃)	Frequency of the MFE structure in the ensemble (%)	ΔG for heterodimer binding (kcal/mol)	Free energy of folding (kcal/mol)
1	siRNA_1	54.2	50.54	‒29.16	0.3
2	siRNA_4	48.9℃	42.73	‒27.42	0.5
					

## Supplementary Materials

Supplementary Table 1. Conditions for rational siRNA design by Reynolds, Amarzguioui, and Ui-Tei algorithms

Supplementary Table 2. BLAST parameters adjusted for off-target filtration

Supplementary Table 3. List of accession numbers of Kaposi's sarcoma-associated herpesvirus strains used in this study

Supplementary Table 4. Consensus sequence generated from the *ORF57* sequences of 76 Kaposi's sarcoma-associated herpesvirus strains using multiple sequence alignment

Supplementary Table 5. List of siRNAs that passed threshold values of selected algorithms

Supplementary Table 6. List of siRNAs that passed conservancy and motif filtration analysis

Supplementary Table 7. List of siRNAs that passed off-target filtration against the human genome

Supplementary Table 8. List of siRNAs that passed filtration steps against miRNA seed region
