# Supplementary material for: An in-silico approach to design potential siRNAs against the ORF57 of Kaposi’s sarcoma-associated herpesvirus
Source: Genomics Inform. 2021 Dec 31;19(4):e47. doi: 10.5808/gi.21057 (PMC8752988; doi:10.5808/gi.21057)
Supplement: Supplementary Table 1. — Conditions for rational siRNA design by Reynolds, Amarzguioui, and Ui-Tei algorithms [file gi-21057-suppl1.pdf]

Supplementary Table 1. Conditions for rational siRNA design by Reynolds, Amarzguioui, and Ui-Tei algorithms

Reynolds rules	Amarzguioui rules	Ui-Tei rules
Presence of A at position 19 of the sense strand<br>Presence of A at position three of the sense strand<br>Presence of U at position ten of the sense strand<br>Absence of G at position 13 of the sense strand<br>Occurrence of three or more A/U base pair at position 15–19 of the sense strand<br>Low internal stability at the target site (Tm 20℃)	Duplex end A/U differential > 0<br>No G at position 19<br>Strong binding of 5´ sense strand<br>No U at position 1<br>Presence of A at position 6<br>Weak binding of 3´ sense strand	A/U at the 5´ termini of the sense strand<br>G/C at the 5´ terminus of the antisense strand<br>At least 4 A/U residues in the 5´ terminal 7 bp of the sense strand<br>No GC stretch longer than 9nt
