# Supplementary material for: An in-silico approach to design potential siRNAs against the ORF57 of Kaposi’s sarcoma-associated herpesvirus
Source: Genomics Inform. 2021 Dec 31;19(4):e47. doi: 10.5808/gi.21057 (PMC8752988; doi:10.5808/gi.21057)
Supplement: Supplementary Table 2. — BLAST parameters adjusted for off-target filtration [file gi-21057-suppl2.pdf]

Supplementary Table 2. BLAST parameters adjusted for off-target filtration

Parameter	Default value	Adjusted value
Database	Nucleotide collection (nr/nt)	Reference RNA sequences(refseq_rna)
Organism	All	Homo sapiens (taxid:9606)
Program selection (optimized for)	Highly similar sequences (megablast)	Somewhat similar sequences (blastn)
Algorithm Parameter		
Max target sequences	100	1000
Expect threshold	0.05	1000
Word size	28	7
Match/Mismatch Scores	1, –2	1, –1
Gap costs	Linear	Existence: 1, Extension: 1
