# Supplementary material for: An in-silico approach to design potential siRNAs against the ORF57 of Kaposi’s sarcoma-associated herpesvirus
Source: Genomics Inform. 2021 Dec 31;19(4):e47. doi: 10.5808/gi.21057 (PMC8752988; doi:10.5808/gi.21057)
Supplement: Supplementary Table 3. — List of accession numbers of Kaposi's sarcoma-associated herpesvirus strains used in this study [file gi-21057-suppl3.pdf]

Supplementary Table 3. List of accession numbers of Kaposi's sarcoma-associated herpesvirus strains used in this study

No. of strain	Accession No.
1	AP017458
2	GQ994935
3	HQ404500
4	JQ619843
5	JX228174
6	KF588566
7	KT271453
8	KT271454
9	KT271455
10	KT271456
11	KT271457
12	KT271458
13	KT271459
14	KT271460
15	KT271461
16	KT271462
17	KT271463
18	KT271464
19	KT271465
20	KT271466
21	KT271467
22	KT271468
23	LC200586
24	LC200587
25	LC200588
26	LC200589
27	MK143395
28	MK733606
29	MK733607
30	MK733608
31	MK733609
32	MN752405
33	NC_009333
34	U75698
35	U93872
36	MK876738.1
37	MK876737.1
38	MK876731.1
39	MT936340.1
40	MT510665.1

41	MT510664.1
42	MT510663.1
43	MN419226.1
44	MN419225.1
45	MN419221.1
46	MN419219.1
47	MK876736.1
48	MK876735.1
49	MK876732.1
50	MT510670.1
51	MT510662.1
52	MT510661.1
53	MT510660.1
54	MT510659.1
55	MT510658.1
56	MT510657.1
57	MT510656.1
58	MN419224.1
59	MN419223.1
60	MK876733.1
61	MT510669.1
62	MT510668.1
63	MT510667.1
64	MT510666.1
65	MT510653.1
66	MT510652.1
67	MT510651.1
68	MT510650.1
69	MT510649.1
70	MT510648.1
71	MN419227.1
72	MN419222.1
73	MN419220.1
74	MT510655.1
75	MT510654.1
76	MK876734.1
