# Supplementary material for: An in-silico approach to design potential siRNAs against the ORF57 of Kaposi’s sarcoma-associated herpesvirus
Source: Genomics Inform. 2021 Dec 31;19(4):e47. doi: 10.5808/gi.21057 (PMC8752988; doi:10.5808/gi.21057)
Supplement: Supplementary Table 4. — Consensus sequence generated from the ORF57 sequences of 76 Kaposi's sarcoma-associated herpesvirus strains using multiple sequence alignment [file gi-21057-suppl4.pdf]

Supplementary Table 4. Consensus sequence generated from the ORF57 sequences of 76 Kaposi's sarcoma-associated herpesvirus strains using multiple sequence alignment

```
ATGGTACAAGCAATGATAGACATGGACATTATGAAGGGCATCCTAGAGGGACTCTGTGTCCTC
CTCTGAGTTTGACGAATCGAGGGACGACGAGACGGACGCACCGACACTGGAAGACGAGCAATT
GTCCGAACCCGCCGAGCCTCCGGCAGACGAGCGCATCCGTGGTACCCAGTCGGCCCAGGGAAT
CCCACCCCCCCTGGGCCGCATCCCAAAAAAATCTCAAGGTCGTTCTCAACTGCGCAGTGAGAT
CCAGTTTTGCTCCCCACTGTCTCGACCCAGGTCCCCCTCACCAGTAAACAGGTACGGTAAAAA
AATCAAGTTTGGAACCGCCGGTCAAAACACACGTCCTCCCCCTGAAAAGCGTCCTCGGCGCAG
ACCACGCGACCGCCTACAATACGGCAGAACAACACGGGGCGGACAGTGTCGCGCTGCACCGAA
GCGAGCGACCCGCCGTCCGCAGGTCAATTGCCAGCGGCAGGATGACGACGTCAGACAGGGTGT
GTCTGACGCCGTAAAGAAACTCAGACTCCCTGCGAGCATGATAATTGACGGTGAGAGCCCCCG
CTTCGACGACTCGATCATCCCCCGCCACCATGGCGCATGTTTCAATGTCTTCATTCCCGCCCC
ACCATCCCACGTCCCGGAGGTGTTTACGGACAGGGATATCACCGCTCTCATAAGAGCAGGGGG
CAAAGACGACGAACTCATAAACAAAAAAATCAGCGCAAAAAAGATTGACCACCTCCACAGACA
GATGCTGTCTTTTGTGACCAGCCGCCACAATCAAGCGTACTGGGTGAGTTGCCGTCGAGAAAC
CGCAGCCGCCGGAGGCCTGCAAACGCTTGGGGCTTTCGTGGAGGAACAAATGACGTGGGCCCA
GACGGTTGTGCGCCACGGGGGGTGGTTTGATGAGAAGGACATAGATATAATTTTGGACACCGC
AATATTTGTCTGCAATGCGTTTGTTACCAGATTTAGATTACTTCATCTTTCCTGCGTTTTTGA
CAAGCAGAGCGAGCTAGCACTGATCAAACAGGTGGCATATTTGGTAGCGATGGGAAACCGCTT
AGTAGAGGCATGTAACCTTCTTGGCGAGGTCAAGCTTAACTTCAGGGGAGGGCTGCTCTTGGC
CTTTGTCCTAACTATCCCAGGCATGCAGAGTCGCAGAAGTATTTCTGCGCGCGGACAGGAGCT
GTTTAGAACACTTCTGGAATACTACAGGCCAGGGGATGTGATGGGGCTACTAAACGTGATAGT
AATGGAACATCACAGCTTGTGCAGAAACAGTGAATGTGCAGCGGCAACCCGGGCCGCAATGGG
GTCGGCCAAATTTAACAAGGGTTTATTCTTTTATCCACTTTCTTAA
```

= 1369 Nucleotides
