# Supplementary material for: An in-silico approach to design potential siRNAs against the ORF57 of Kaposi’s sarcoma-associated herpesvirus
Source: Genomics Inform. 2021 Dec 31;19(4):e47. doi: 10.5808/gi.21057 (PMC8752988; doi:10.5808/gi.21057)
Supplement: Supplementary Table 5. — List of siRNAs that passed threshold values of selected algorithms [file gi-21057-suppl5.pdf]

Supplementary Table 5. List of siRNAs that passed threshold values of selected algorithms

Start positions	Sense strand sequence	Antisense strand sequence	% GC	Reynolds	Amarzguioui	Ui-Tei	i-Score	Sfold total score	Conservancy
294	CAGUAAACAGGUACGGUAA	UUACCGUACCUGUUUACUGgu	42.1	6	5	Ib	76.3	12	100%
391	CCUACAAUACGGCAGAACA	UGUUCUGCCGUAUUGUAGGcg	47.4	6	4	Ib	68.9	15	80.26% (61/76)
392	CUACAAUACGGCAGAACAA	UUGUUCUGCCGUAUUGUAGgc	42.1	8	3	Ia	69.5	17	97.37% (74/76)
511	CGCCGUAAAGAAACUCAGA	UCUGAGUUUCUUUACGGCGuc	47.4	6	4	Ib	72	12	98.68% (75/76)
664	GGAUAUCACCGCUCUCAUA	UAUGAGAGCGGUGAUAUCCcu	47.4	8	4	Ia	77.5	14	100%
694	CAAAGACGACGAACUCAUA	UAUGAGUUCGUCGUCUUUGcc	42.1	8	4	Ia	76	15	100%
700	CGACGAACUCAUAAACAAA	UUUGUUUAUGAGUUCGUCGuc	36.8	8	5	Ia	84.7	17	100%
972	CCAGAUUUAGAUUACUUCA	UGAAGUAAUCUAAAUCUGGua	31.6	7	3	Ia	75.9	13	100%
1068	GCUUAGUAGAGGCAUGUAA	UUACAUGCCUCUACUAAGCgg	42.1	6	4	Ia	76.6	13	100%
1187	GGACAGGAGCUGUUUAGAA	UUCUAAACAGCUCCUGUCCgc	47.4	7	3	Ia	72.3	13	97.37% (74/76)
1329	CCAAAUUUAACAAGGGUUU	AAACCCUUGUUAAAUUUGGcc	31.6	6	4	Ib	68.1	12	100%
1330	CAAAUUUAACAAGGGUUUA	UAAACCCUUGUUAAAUUUGgc	26.3	7	3	Ib	74.6	12	100%
