# Supplementary material for: An in-silico approach to design potential siRNAs against the ORF57 of Kaposi’s sarcoma-associated herpesvirus
Source: Genomics Inform. 2021 Dec 31;19(4):e47. doi: 10.5808/gi.21057 (PMC8752988; doi:10.5808/gi.21057)
Supplement: Supplementary Table 6. — List of siRNAs that passed conservancy and motif filtration analysis [file gi-21057-suppl6.pdf]

Supplementary Table 6. List of siRNAs that passed conservancy and motif filtration analysis

Name	Start positions	Sense strand sequence	Antisense strand sequence
siRNA_1	294	CAGUAAACAGGUACGGUAA	UUACCGUACCUGUUUACUGgu
siRNA_2	664	GGAUAUCACCGCUCUCAUA	UAUGAGAGCGGUGAUAUCCcu
siRNA_3	694	CAAAGACGACGAACUCAUA	UAUGAGUUCGUCGUCUUUGcc
siRNA_4	700	CGACGAACUCAUAAACAAA	UUUGUUUAUGAGUUCGUCGuc
siRNA_5	972	CCAGAUUUAGAUUACUUCA	UGAAGUAAUCUAAAUCUGGua
siRNA_6	1068	GCUUAGUAGAGGCAUGUAA	UUACAUGCCUCUACUAAGCgg
