# Supplementary material for: An in-silico approach to design potential siRNAs against the ORF57 of Kaposi’s sarcoma-associated herpesvirus
Source: Genomics Inform. 2021 Dec 31;19(4):e47. doi: 10.5808/gi.21057 (PMC8752988; doi:10.5808/gi.21057)
Supplement: Supplementary Table 7. — List of siRNAs that passed off-target filtration against the human genome [file gi-21057-suppl7.pdf]

**Supplementary Table 7.** List of siRNAs that passed off-target filtration against the human genome

| Name | Start positions | Sense strand sequence | Antisense strand sequence | BLAST pass Sense strand | BLAST pass Antisense strand | Overall BLAST pass |
|---|---|---|---|---|---|---|
| siRNA_1 | 294 | CAGUAAACAGGUACGGUAA | UUACCGUACCUGUUUACUGgu | Yes | Yes | Yes |
| siRNA_2 | 664 | GGAUAUCACCGCUCUCAUA | UAUGAGAGCGGUGAUAUCCcu | Yes | Yes | Yes |
| siRNA_3 | 694 | CAAAGACGACGAACUCAUA | UAUGAGUUCGUCGUCUUUGcc | No (17/18) | No (17/18) | No |
| siRNA_4 | 700 | CGACGAACUCAUAAACAAA | UUUGUUUAUGAGUUCGUCGuc | Yes | Yes | Yes |
| siRNA_5 | 972 | CCAGAUUUAGAUUACUUCA | UGAAGUAAUCUAAAUCUGGua | Yes | Yes | Yes |
| siRNA_6 | 1068 | GCUUAGUAGAGGCAUGUAA | UUACAUGCCUCUACUAAGCgg | No (17/19) | No (17/19) | No |
