# Supplementary material for: An in-silico approach to design potential siRNAs against the ORF57 of Kaposi’s sarcoma-associated herpesvirus
Source: Genomics Inform. 2021 Dec 31;19(4):e47. doi: 10.5808/gi.21057 (PMC8752988; doi:10.5808/gi.21057)
Supplement: Supplementary Table 8. — List of siRNAs that passed filtration steps against miRNA seed region [file gi-21057-suppl8.pdf]

Supplementary Table 8. List of siRNAs that passed filtration steps against miRNA seed region

Name	Start positions	Sense strand	Antisense strand	Overall BLAST Pass	miRNA seed matching
siRNA_1	294	CAGUAAACAGGUACGGUAA	UUACCGUACCUGUUUACUGgu	Yes	Pass
siRNA_2	664	GGAUAUCACCGCUCUCAUA	UAUGAGAGCGGUGAUAUCCcu	Yes	No (matched with a human miRNA seed)
siRNA_4	700	CGACGAACUCAUAAACAAA	UUUGUUUAUGAGUUCGUCGuc	Yes	Pass (matched with a mouse miRNA seed)
siRNA_5	972	CCAGAUUUAGAUUACUUCA	UGAAGUAAUCUAAAUCUGGua	Yes	No (matched with three human miRNA seed)
